# The Paradoxical Relationship Between Health Promotion and the Social Media Industry

**DOI:** 10.1177/15248399211064640

**Published:** 2021-12-29

**Authors:** Marco Zenone, Nora Kenworthy, Skye Barbic

**Affiliations:** 1 London School of Hygiene and Tropical Medicine, London, UK; 2 University of Washington Bothell, Bothell, WA, USA; 3 University of British Columbia, Vancouver, British Columbia, Canada

**Keywords:** adolescent, social media, commercial determinants of health, mental health

## Abstract

Mounting evidence suggests that problematic adolescent social media use is associated with poor mental health. To respond to increased adolescent mental health concerns, health promoters increasingly rely on social media initiatives to promote their resources, programs, and services. This creates a paradoxical situation where social-media-linked adverse mental health outcomes are addressed using the same tools and platforms that can contribute to the development of such issues. It also highlights several areas of needed critical assessment in health promotion usage of social media platform features and products, such as addictive platform design, targeted marketing tools, data collection practices, impacts on underserved groups, and conflicts of interest. To advance subsequent action on these tensions, we offer three recommendations for health promoters that build upon existing scholarship and initiatives, including adapting ethical guidelines for health promoters using social media, adopting conflicts of interest policies, and promoting interdisciplinary scholarship.

To respond to increased adolescent mental health concerns, health promoters increasingly rely on social media platforms and initiatives to promote their resources, programs, and services. However, while social media offers useful tools for health promoters to reach young people, social media platforms’ business practices—such as targeted advertising, unauthorized data collection, and the addictive traits of platform design (Montag et al., 2019)—raise mounting concerns. Given these tensions, health promoters should practice greater scrutiny and awareness of the potential harms of social media platforms and critically assess the continued reliance on social media in health promotion practice.

Recent reviews have found associations between youth social media use and mental health concerns, including depression, anxiety, and psychological distress (Keles et al., 2020). Researchers increasingly point to time spent on social media contributing to increased risk of mental health symptoms and poor well-being (Naslund et al., 2020). The design and user experience of major social media platforms has been shown to be designed to elicit addictive behaviors (Montag et al., 2019). However, research also shows that people respond to and engage with technology in a wide variety of ways, and thus the harms (and benefits) of technology may not be inevitable, or evenly dispersed across populations (Owens & Lenhart, 2020). Retaining the focus and ongoing attention of the user is a primary goal of social media platforms because it allows the auctioning and subsequent profit from advertisements shown to the user. Content that engages the user is predicted using artificial intelligence designed to maximize screen time, as well as other strategies, such as a variety of notifications (e.g., updating the user a friend has recently shared a post), or suggesting content with limited moderation of potential harms, such as misleading or false information. Although users may try to modulate their use of social media to protect their mental health, they have little control over advertisements that are shown to them on these platforms, which can include harmful content, or advertisements that promote health-harming products. These tactics have largely been successful and profitable. In 2019, Facebook reported earning US$29.92 billion from advertising revenue in the United States, while Instagram earned US$9.45 billion and Twitter earned US$1.57 billion (Guttmann, 2020).

Conversely, to respond to increased mental health challenges and awareness among adolescents and young adults, public health and health promotion practitioners are increasingly delivering interventions through social media and relying on such platforms to advertise their resources and supports. For example, Foundry, a province-wide network of integrated youth care centers in British Columbia, Canada, designed a social media campaign connecting boys and young men to mental health supports (Zenone et al., 2020). The digital environment offers benefits to reach underserved groups. Many young people report using the digital space to foster community and for timely access to health and social services that are youth-centered, accessible, and aligned with their principles and values (Yonker et al., 2015). The internet can also be a particularly important place for community and empowerment for marginalized groups (Bishop et al., 2004).

This creates a paradoxical situation where the tools for mental health intervention, particularly among young people, rely on the same social media platforms and mechanisms that can contribute to adverse mental health outcomes. Similarly, social media companies simultaneously profit from platform designs that facilitate addictive behaviors to retain focus for targeted advertisements, and also from paid advertisements by health promotion organizations that seek to connect people to mental health or other resources. This situation poses a challenge to health promotion and public health values. Health promotion at its core is about tackling upstream, structural issues; addressing the harms of social media while relying on, and contributing to, the incentive structures and profit motives of those platforms makes this difficult. The *Ottawa Charter for Health Promotion* states, “It [health promotion] puts health on the agenda of policy makers in all sectors and at all levels, directing them to be aware of the health consequences of their decisions and to accept their responsibilities for health” (World Health Organization, 1986). The Charter provides an opportune moment to reconsider the relationship between health promotion, public health, and social media. Moreover, in the past, health promotion has provided leadership in determining the appropriate role and cautions of social media in public health (Korda & Itani, 2013; Neiger et al., 2012). Given the rapid pace of social media platform market expansion, health promotion scholars must continue to critically engage with social media as a determinant of health and an important channel for their work.

There are several key tensions where health promotion can critically assess its relationship with social media companies. First, it is likely that the efficiency in health promotion advertising investment and subsequent advertisement views may be enhanced in part from addictive platform design (Montag et al., 2019) which retains the attention of users. Second, the targeted advertisement features that assist in reaching underserved populations may be collected without informed consent of social media users. For instance, Facebook can create “custom audiences” which advertisers can target based on the activity of their social profile, such as what pages they like, how often or when they are on their device, or what content they engage regularly (Facebook Business, 2021). Users may not be aware this information is collected and analyzed by Facebook to support advertising features. Related to this point, health-harming industries, such as ultra-processed foods, may make use of the same tools offered to health promoters, including to market harmful products to children. Third, social media evaluation metrics—such as engagements, likes, or comments—have become normalized metrics of success in health promotion evaluation and to justify funding (Neiger et al., 2012). However, the data collected are arguably collected from means that would be unethical in non-online contexts due to privacy and data use concerns, especially among young people. Social media analytics provides insights into the demographic and activity profile of persons engaging with content, including age, gender, city, language, and time spent on page. The collection of this information in other offline settings may require research ethics or parental consent. Fourth, at this time, social media advertising fails to acknowledge the differential harms of technology to specific racial groups, for whom social media offers far fewer safe spaces, amplifies racist rhetoric, and often fails to protect users from actual harms when concerns are raised (Daniels, 2012; Zang, 2021). Without acknowledgment, and action, health promotion will not serve these groups and may reinforce inequities. Last, social media platforms are for-profit entities with financial motives, and public health and researchers increasingly rely upon partnerships with them to gather data and reach the public (Kenworthy et al., in press). Health promotion professionals need clearer guidelines for what constitutes conflicts of interest in these areas, particularly for the purposes of partnership development or research reporting.

To advance critical discussion and subsequent action on these tensions, we offer three recommendations for health promoters that build upon existing scholarship and initiatives. First, we call for the development of ethical guidelines in health promotion practice as it relates to social media. Existing ethical guidelines, such as from the Association of Internet Research (AOIR, 2021)—a nonprofit, independent research group—can form a strong, adaptable starting point. Second, we encourage health promotion journals, professional organizations, and practice groups, to adopt conflicts of interest policies and declarations when working with social media platforms in any capacity. Existing conflicts of interest mechanisms, such as the Guidelines International Network principles (Schünemann et al., 2015), can be expanded upon to capture interactions with social media platforms, usage of their data, and funding. Next, we encourage interdisciplinary collaboration from numerous public health disciplines, such as the commercial determinants of health (CDOH), to leverage experience and knowledge in confronting social media as not only a public health tool but a corporate actor with significant power and influence over public health practice. We also encourage interdisciplinary collaboration to address social media industry’s business practices and subsequent harms, including addictive platform design, targeted marketing, and data collection concerns. In conclusion, advancing the mental health of young people in the social media age requires implementing safer social media practices and acting upon the tensions between social media advertising and health promotion.
